# Supplementary figures and images for: The vaginal Torquetenovirus titer varies with vaginal microbiota composition in pregnant women
Source: PLoS One. 2022 Jan 20;17(1):e0262672. doi: 10.1371/journal.pone.0262672 (PMC8775304; doi:10.1371/journal.pone.0262672)

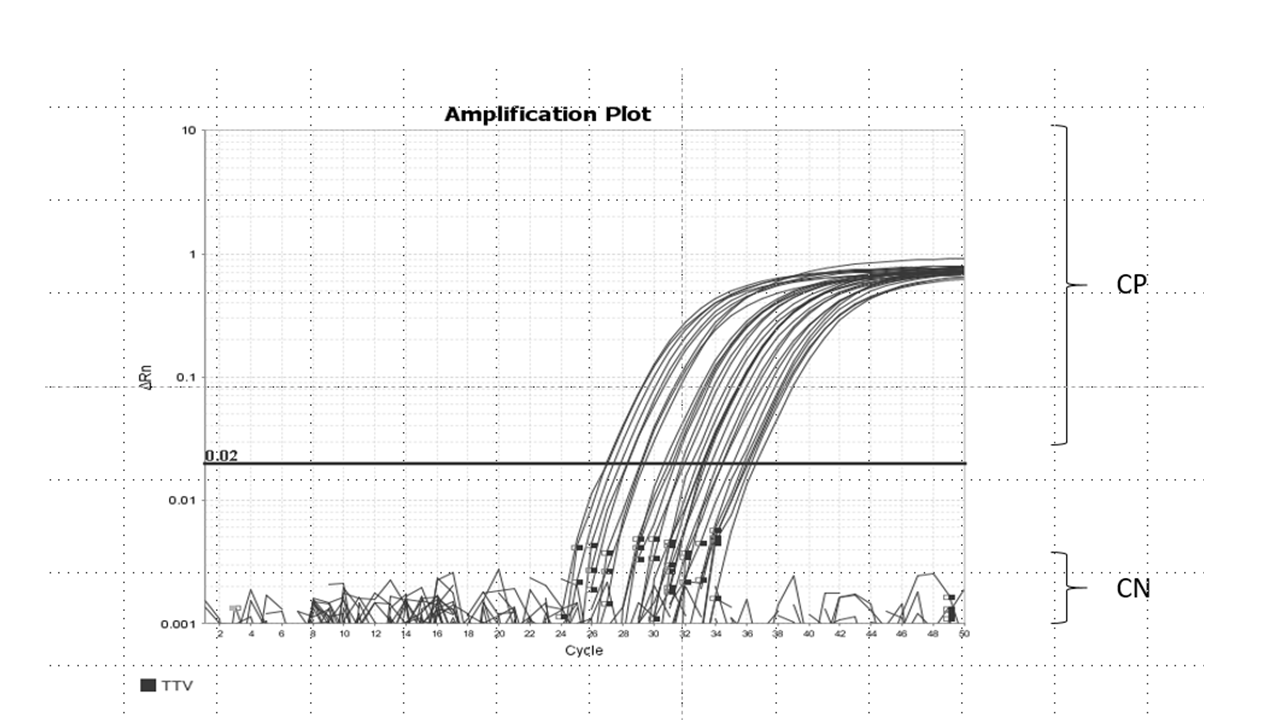

Supplement: S1 Fig — The TTV titer was obtained by using a qPCR based on the TaqMan™ Universal PCR master mix protocol (Thermo Fisher Scientific, Warrington, UK), a purified DNA template (~100 ng/reaction) in a final volume of 25μl. The condition for thermocycling was 50oC for 2 minutes, 95oC for 15 seconds and followed by 50 cycles of 95oC for 15 seconds and 60oC for 1 minute, in a Quantstudio™ 5 thermocycler. The data were analyzed using QuantStudio Design & Analysis Software v.1.4.1. The analytical sensibility (LOD>95%) of TTV qPCR is 40 copies/ml. Cycle number is shown on the x-axis and change in florescent intensity (ΔRn) is shown on the y-axis; the horizontal line indicates the threshold setting. NC, Non-template control or Negative control; CP, TTV positive genital fluid samples. (TIF) [file pone.0262672.s001.tif]
