# Supplementary material for: The vaginal Torquetenovirus titer varies with vaginal microbiota composition in pregnant women
Source: PLoS One. 2022 Jan 20;17(1):e0262672. doi: 10.1371/journal.pone.0262672 (PMC8775304; doi:10.1371/journal.pone.0262672)
Supplement: S1 Table — (PDF) [file pone.0262672.s002.pdf]

S1 Table. Association between the TTV titer in vaginal secretions and the dominant bacterium in the vaginal microbiome

Dominant bacterium	No. women	Median $\log_{10}$ TTV titer (Interquartile range)[a]
L. crispatus	206	1.6 (<1.0, 4.4)[b]
L. iners	159	4.1 (<1.0, 5.1)
L. jensenii	16	0.0 <1.0, 4.8)
L. gasseri	12	1.5 (<1.0, 5.3)
L. delbrueckii	1	<1.0
L. vaginalis	1	<1.0
G. vaginalis	59	4.4 (<1.0, 5.5)
Leptopiraceae	4	5.2 (3.8, 6.0)
None	36	4.4 (3.4, 5.0)

Dominant bacterium indicates >50% of the total bacteria identified. None indicates samples in which no bacterium was present at >50%.

[a]Differences in TTV titer when different bacteria dominate p = 0.0001 (Kruskal-Wallis test)

[b]p = 0.0001 vs. *L. iners*, 0.0009 vs. None, 0.0016 vs. *G. vaginalis* (Mann-Whitney test).
