# Supplementary material for: The vaginal Torquetenovirus titer varies with vaginal microbiota composition in pregnant women
Source: PLoS One. 2022 Jan 20;17(1):e0262672. doi: 10.1371/journal.pone.0262672 (PMC8775304; doi:10.1371/journal.pone.0262672)
Supplement: S2 Table — (PDF) [file pone.0262672.s003.pdf]

S2 Table. Association between TTV titer in vaginal secretions and race

Race	No. women	Median TTV titer (Interquartile range)
White	230	3.7 (<1.0,5.0)
Mixed	195	3.7 (<1.0, 5.1)
Black	37	4.2 (<1.0, 5.0)

p = 0.9183 (Kruskal-Wallis test)
