# Supplementary material for: The vaginal Torquetenovirus titer varies with vaginal microbiota composition in pregnant women
Source: PLoS One. 2022 Jan 20;17(1):e0262672. doi: 10.1371/journal.pone.0262672 (PMC8775304; doi:10.1371/journal.pone.0262672)
Supplement: S3 Table — (PDF) [file pone.0262672.s004.pdf]

S3 Table. TTV titer by site

Site	No. samples	Median TTV titer (Interquartile range)
Fortaleza	50	2.6 (<1.0, 4.6)
Jundaia	148	3.8 (<1.0, 5.2)
Sao Paulo	279	3.7 (<1.0, 5.0)

p = 0.2941 (Kruskal-Wallis test)
