# Supplementary material for: The vaginal Torquetenovirus titer varies with vaginal microbiota composition in pregnant women
Source: PLoS One. 2022 Jan 20;17(1):e0262672. doi: 10.1371/journal.pone.0262672 (PMC8775304; doi:10.1371/journal.pone.0262672)
Supplement: Attachment — Submitted filename: 141121_TTV PLoS response to reviewers.docx [file pone.0262672.s005.docx]

**PONE-D-21-30550**

**The vaginal Torquetenovirus titer varies with vaginal microbiome composition in pregnant women**

Dr. Caroline Mitchell

Academic Editor, PLoS One

Dear Dr. Mitchell,

We thank you and the reviewers for the favorable review and constructive comments on our manuscript. We have incorporated all of the suggestions into our revision. The specific comments and our responses are as follows:

**Editor**.

I strongly agree with the reviewers' comments that the conclusions are overstated, and several speculative comments are made which are not supported by the data. In a field like this where data are few, I would encourage you to make more factual statements about the findings about correlation between TTV titer and microbial communities, and not speculate about the causal relationship between them - especially based on cross-sectional data.  It might be worth a little more discussion of TTV and its link to immune competence, and how you think TTV could be used as a biomarker IF your results are confirmed.

Answer: The manuscript has been extensively revised so that our conclusions and other statements are more in keeping with the data and are not overly speculative. The Introduction has been expanded to include more information and references on TTV, especially as related to pregnancy.

Line 64: Goal is to determine “…if the TTV titer in vaginal secretions was predictive of vaginal microbiome composition and pregnancy-related parameters.”

Answer: This sentence has been deleted and replaced with a more specific aim.

Line 90: “All samples were suitable for DNA amplification by using internal control.” – it is unclear exactly what this means? Was a region amplified to show there was no inhibition? Was the quality and quantity of nucleic acid measured?

Answer: This is now fully explained in lines 104-106.

Line 180-185: “We propose that a low or undetectable vaginal TTV titer is a biomarker for lower genital tract quiescence during the second trimester of pregnancy, similar to when L. crispatus is dominant at that site.” – How can you separate the quiescence and L. crispatus dominance? It doesnt' seem that you have shown that the two are separately associated with TTV.

Answer: We agree that this is an example of our over-interpretation of the data. This has been replaced by a more limited and more focused evaluation of the data in the revised manuscript.

Line 191-193: “Thus, a low vaginal TTV titer appears to parallel L. crispatus vaginal dominance as an indicator of reproductive tract quiescence in pregnant women.” This is a very robust statement that doesn’t necessarily seem supported by the existing data.

Answer: We agree and have deleted this sentence and replaced it with a more focused interpretation in the revised manuscript.

Line 199-201: “The findings also strongly suggest that under conditions when vaginal TTV titers are elevated, nutritional requirements in the vaginal milieu are altered such that L. crispatus is no longer capable of preferential proliferation.” There is no evidence to support this claim - it is very speculative, and should be removed or rephrased.

Answer: This speculative sentence has now been deleted in the revised manuscript.

Based on the fact that TTV likely infects lymphoid cells, and that lower immune activation is seen with low-diversity Lactobacillus dominant communities, could it not be equally likely that as the immune system is activated, more lymphocytes arrive at the mucosa and more TTV? So higher TTV is a marker of immune activation, but not at all part of the causal pathway.

Answer: Again, we completely agree with your interpretation and now specifically state this in Discussion.

Could the authors explain why would it be better to measure TTV than anything else? What does using TTV as a biomarker gain for the scientist or clinician?

Answer: We do not state that TTV is superior to measurement of the individual bacteria in the vaginal microbiome or to quantitation of vaginal compounds. We now state, based on conclusions from prior TTV-related studies, that the observed associations between TTV and vaginal microbiota and vaginal compounds provides new insights into alterations in the vaginal environment that are present when different bacteria predominate.

**Reviewer #1**
The paper is a large study examining the correlation between the vaginal microbiome and Torquetenovirus, matrix metalloproteinases, tissue inhibitors of MMP and lactic acid isomers in 496 pregnant women. The main strength is the size of the cohort, and that it was spread over 3 sites. The results are simple and the conclusions concise. Therefore, the study provides substantial evidence of the inverse link between L. crispatus and TTV titer in the vaginal cavity.

Answer: We thank the reviewer for this positive assessment.

In my opinion, the greatest weakness of the paper is a tendency towards over over-interpretation of results. It makes little case for why should be TTV titer should be a superior biomarker to L. crispatus. Since the discussion exclusively discusses the link between L. crispatus and decreased immune activation which is thought to correlate to TTV, it is not clear a qPCR for L. crispatus would not be a much more direct measurement. Similarly, the D-lactic acid and TMP production directly tied to L. crispatus dominance/minority and therefore appear almost redundant. Without measurements of immunological markers, the data only seems to conclude that TTV titter can be used to discriminate a L. crispatus dominated vaginal microbiome from one dominated by G. vaginalis, L. iners or no specific species. I am not sure that the claim in abstract that TTV reflects local conditions can be substantiated, since all measured substance are bacterial metabolites.

Answer: As stated above we have now greatly modified our interpretations of the actual data. We must disagree with the reviewer as to the origin of MMPs and TIMPs. These compounds are of host origin; only the D- and L-lactic acid are bacterial. Nevertheless, we have modified our conclusion as stated in our last comment to the Editor.

Line 51: Reference needed. Answer: We have included several references that describe the relative abundance of components of the vaginal microbiota in this revised version.

Line 61: I would prefer that you place references right after the relevant text section, rather than grouped together at the end of the section. Also relevant for many other citations in manuscript. Answer: We have rearranged the references as recommended.

Line 75: Do you have any approval registration numbers etc.? The reader should be able to verify the approval based on the information in the manuscript. Answer: We now list in Methods specific details of the Institutional Review Board approval.

Line 90: I cannot make sense of this sentence. What internal control are you referring to? Answer: We apologize for this unclear sentence. We have now greatly clarified our protocol and included a reference in the revised manuscript.

Line 106: Even though the methods have been described previously, the reagent kits and sequencing platform should be briefly described for ease of reading. Answer: This has now been added in the revised manuscript.

Line 146: Table 2 was quite unintuitive for me to understand. You compare the L. iners TTV titers to those of other bacteria and find that some are statistically significant from only three of the other dominant bacteria, but this is initially very clear from the figure. However, in the text you write about the positive and negative correlations of bacteria with TTV titer, while Table 2 compares L. iners other bacteria, which is slightly confusing. Possibly a bar plot showing significant pairwise differences would be a better way to present this, while also giving a better idea of variance. Answer: Thank you for this comment. We have significantly shortened the Table by combining bacterial species with similar known activities to now make the observed differences between species more easily understandable. We have also expanded the text to include more information in the lines 144 -170.

Line 151: The role of MMP’s should be briefly described in the introduction. Answer: This has now been added to the Introduction in the revised manuscript.

Line 165: Were there any correlations between dominant vaginal bacteria and patient characteristics? Answer: Since most women at risk for preterm birth were treated with progesterone, as stated in the text, there were no observed differences between bacterial dominance and patient characteristics or outcome. We have now added a statistical analysis that demonstrates no associations between TTV titer and patient characteristics.

Line 177: You write presence/abundance, but in the results section you exclusively use dominant species which is based on abundance. I think presence could be a bit misleading, since the word normally refers to binary presence/absence metrics used in community analysis. Answer: We agree and have now removed the word “presence” from the manuscript.

Line 189: I am not sure that you can assume anything about quiescence, as you do not measure immune response or inflammatory markers at all. If you insist on coupling TTV titer with quiescence, it is necessary to include literature references strongly correlating L. iners domination with quiescence. Answer: We agree that use of the word “quiescence “ is speculative and it has now been removed from the text.

Line 199: This paragraph is quite vague in its current for and should either be expanded or omitted. Answer: We have now omitted this speculation from Discussion.

The introduction should have some rationale for why you use L. iners as a reference instead of some other vaginal bacteria. To my knowledge a L. iners dominated vaginal bacterial community is not the only healthy vaginal community state type. Answer: The Introduction has been extensively modified to clarify what is known about different bacteria in the vaginal microbiota. Actually, *L. iners* dominance is associated with an unhealthy pregnancy outcome.

Citations should be placed before the full stop consistently. Answer: This has now been corrected.

It should be noted in the discussion that the low sample counts for L. jensenii, gasseri, delbrueckii, vaginalis and Leptopiraceae could be reason that other bacteria are not significantly different. From the median TTV titers, it appears that L. jensenii and gasseri could have a similar inverse correlation as L. crispatus. Answer: Thank you for this suggestion that we have now incorporated into study limitations in Discussion. Table 2 has also been modified in response to your suggestion.

I think that the focus of the introduction should be shifted from immune quiescence towards vaginal microbiome, since that is what is measured in the study. Answer: We agree and have extensively modified the Introduction to now deal mostly with TTV and with the vaginal microbiota.

I am unable to find link to the original raw data files from the study, despite this being stated for the submission. I have only had access to the main manuscript file for review, so I apologize if this information is included as supplementary material. Answer: The raw data files are available upon request.

The study definitely has merit, but the conclusions need to be toned down significantly, and table 2 data should be presented more clearly. Answer: Thank you for acknowledging the merit of our study. We have toned down the conclusions considerably and modified Table 2.

**Reviewer #2**

Tozetto-Mendoza et al. describe the vaginal TTV loads in relationship with microbiome composition in pregnant women. The manuscript is of some interest providing new data on a largely unknown topic. Overall, the data are scientifically sound and concisely and clearly described. Answer: We thank the reviewer for his kind comments.

**Reviewer #3**

-line 25: missing word: if ‘it’ was predictive. Answer: This has been corrected.

-line 26: Did the authors assess the microbiome or microbiota? Answer: Throughout the text microbiome has now been replaced by microbiota.

-line 29: definition dominance unclear: does this refer to relative abundance?. Answer: We now specify that dominance refers to relative abundance and this term is now used throughout the text.

-line 36: conclusion unclear – the study describes associations, directionality of these associations and causality cannot be addressed by its design. Answer: We agree and have extensively modified the discussion and the conclusion in this revised version.

Introduction
-The referencing style is inconsistent: e.g line 46 individuals.[1,2] vs. line 49 diseases [6,7]. Answer: The reference style is now uniform throughout the manuscript.

- line 45: *Alphatorquetenoviru*s should be italicized. Answer: This has been done.

- it might be helpful to add a paragraph on (i) how TTV are acquired since there’s some literature describing placental acquisition, (ii) where TTV replicates as application appears to occur in T cells which is relevant to the vaginal site and to emphasise (iii) that TTV has previously been suggested as marker of immune status/suppression. Answer: The Introduction has been modified to include more information about TTV as requested.

-line 50/51: Please add references to support this statement and state for which geography this statement is true. In various parts of the world, pregnancy microbiota composition seems to differ considerably. Answer: This statement has been modified and references added.

-line 56-58: Please add references to support this statement. Answer: References have been rearranged to more appropriate places in the manuscript.

-line 62-63: Please expand on this section and include other relevant literature, e.g. [https://doi.org/10.1086/319983](https://urldefense.proofpoint.com/v2/url?u=https-3A__doi.org_10.1086_319983&d=DwMFaQ&c=lb62iw4YL4RFalcE2hQUQealT9-RXrryqt9KZX2qu2s&r=GkVeazppdM1GOrt42pvg0z0w4N7OfqswaK2i1TXhaQ4&m=rurOI1htORavTd7jwALq1waSNT9NIVQbOzr0zbMkhlGwKWHsLkVxxM_ngpVNExyk&s=QWdRJfKsipRnFy9VFOaxKA9Q9VB71MukDyNCulM4GXM&e=); [https://doi.org/10.1159/000348514](https://urldefense.proofpoint.com/v2/url?u=https-3A__doi.org_10.1159_000348514&d=DwMFaQ&c=lb62iw4YL4RFalcE2hQUQealT9-RXrryqt9KZX2qu2s&r=GkVeazppdM1GOrt42pvg0z0w4N7OfqswaK2i1TXhaQ4&m=rurOI1htORavTd7jwALq1waSNT9NIVQbOzr0zbMkhlGwKWHsLkVxxM_ngpVNExyk&s=_3MmK0N78i7qGFKWZ6Oe7bsYDMd25G2l7gKhHm_TagI&e=); PMID: 12082401 and others. Answer: The suggested reference plus others on TTV in pregnancy have been added.

-line 65: Clarify rational of the study as it can only describe associations due to it’s cross-sectional design. Answer: We agree and have modified and now more clearly describe the aims of the study.

Methods
-line 69: it might be helpful to add the gestational age range. Answer: This is provided in Table 1.

-line 91- controls? Answer: This has now been extensively clarified and a new reference has been added.

-line 103: please change ‘microbiome’ to ‘microbiota’ – this should be corrected throughout the manuscript, as appropriate. Answer: This has been done as suggested.

-line 106: Please add the public registry and reference number for the project to indicate where the sequencing data is publicly available. Answer: References are provided in the text for the sequences used in our study. None of the sequencing data is original but has been used numerous times by other investigators.

-statistics: where analyses adjusted for multiple comparisons? If so, how? Answer: The analyses were not adjusted for multiple comparisons since the number of comparisons is small and the p values in all comparisons were highly significant.

-line 113: There seems to be a word missing in the last sentence. Please add which Graphpad version was used. Answer: This has now been added.

Results
-line 116 etc: please add SD or IQR, as appropriate. Answer: This information is provided in the Table and it seemed to be redundant to also include it in the text.

-line 120: missing comma after available. Answer: This has been corrected.

-Table 1: Is this the BMI at the visit, or pre-pregnancy BMI? I am uncertain how this is relevant to the current study. Answer: BMI refers to BMI at the time of the second trimester visit. Its inclusion is just to provide a more complete characterization of the patients.

-line 132: microbiota. Answer: This has been changed.

-line 133: clarify the definition of the dominant bacteria: I assume this is based on relative abundance? Were taxa merged on the lowest taxonomic rank that was available, or species level?
-In what proportion of samples was TTV detectable? Have the authors performed analyses based on presence vs. absence of TTV? Answer: We have now clarified that dominant bacteria refers to the situation where the relative abundance of a specific bacterium was >50% of the total number of bacteria identified. We have modified Table 2 to now also present data on the species level. We have now included a sentence in Results describing the percent of cases in which the TTV level was below our lower limit of detection. We have not performed a separate analysis based on the detection or non-detection of TTV. We did not think this was would provide additional valid information since it would eliminate a different percentage of values for each bacterium.

-It might be nice to present the results from Tables 2 and 3 visually, rather than as table. P-values could then be added to the graph, and all p-values should be adjusted for multiple comparisons. Answer: As mentioned in our comment to Reviewer 1 Table 2 has been extensively revised to make the data more easily understandable.

-line 141-143: Please report the Spearman rho and not only the p-value. Answer: This has now been added as requested.

-line 154: comma missing. Answer: This has been added.

-line 165-170: Could these data be added as supplementary data? Where there any differences by site? How did the concentrations of measured MMPs etc relate to the dominant bacterial taxa?

Answer: We have now added a statistical analysis of all these data.

Discussion
-the font type is not consistent throughout the discussion.

Answer: This has been corrected.

General comments:

This is an interesting and relevant study that will be of interest to researchers in the field. I have some concerns about the statistical analysis, e.g. the lack of adjusting p-values for multiple comparisons, and the conclusion the authors make based on the available data. It should be emphasised that the authors describe associations and cannot make any conclusions about directionality of events or causality. A thorough proof-reading of the manuscript might be advisable to reduce grammatical errors. Data that is not shown in the manuscript should be included as supplementary material.

Answer: We have addressed all the issues mentioned by the reviewer and the text has been significantly modified to reduce grammatical errors and reduce the amount of speculation.

We are available to send you any further information you may require.

Yours faithfully,

Tania Regina Tozetto-Mendoza and co-authors

*Laboratory of Virology,*

*Institute of Tropical Medicine*

*School of Medicine, University of São Paulo, Brazil*
