# Supplementary material for: The vaginal Torquetenovirus titer varies with vaginal microbiota composition in pregnant women
Source: PLoS One. 2022 Jan 20;17(1):e0262672. doi: 10.1371/journal.pone.0262672 (PMC8775304; doi:10.1371/journal.pone.0262672)
Supplement: Attachment — Submitted filename: TTV PLoS One response to reviewers 12_21.docx [file pone.0262672.s006.docx]

Academic Editor

Dear Dr. Mitchell,

We thank you and the reviewers for helpful comments on our revised submission, ONE-D-21-30550R1 “The vaginal Torquetenovirus titer varies with vaginal microbiota composition in pregnant women”. The individual comments and are responses are detailed below.

1. I agree with the reviewer's comments about the difficulty in assessing association with pregnancy outcomes when some people were treated with progesterone, but we have no information about who those people were. If discussing pregnancy outcomes, please present metadata and TTV data that allow more robust interpretation. Table 1 could be presented with 3 columns: term, sPTB, iPTB and could include those data.

**Response:** We completely agree with your comment and those of the reviewer that we are not in a position to accurately evaluate the association between TTV titer and pregnancy outcome. Providing progesterone treatment to women deemed to be at high risk for a preterm birth introduced another variable (uncontrolled) that made conclusions about a relationship between vaginal TTV titer in the second trimester and risk of premature delivery in our population difficult to interpret. We acknowledge that the relation between TTV and premature delivery must of necessity be studied separately in a prospective investigation in which all subjects receive the same treatment. Therefore, in our revision we have now removed as an aim of our study to determine the association between TTV and pregnancy outcome. However, we have now added data in Results showing that there is no difference in the mid-trimester TTV titer between women who were subsequently treated with progesterone (and therefore deemed to be at elevated risk for preterm labor) and untreated women (not at high risk). This is a more accurate statement of what we actually determined. We would like to stress that the vaginal samples from all subjects were obtained **prior** to progesterone administration, ensuring that this procedure did not influence the vaginal TTV titer, microbiome composition or the concentration of compounds in vaginal fluid. The remaining aims of the study and our conclusion in defining associations between TTV titer, the dominance of individual bacteria in the vaginal microbiota and concentrations of compounds in vaginal fluid remain valid and we believe are unique and worthy of dissemination.

**Reviewer’s Comments to the Author**

Reviewer #1: I think that the authors have done a good job, and that the revised manuscript has been greatly improved by the streamlining and more substantiated conclusions. There are still 2 points that I think should be addressed before acceptance:
1) All supplementary material should be uploaded as supplementary files along with the manuscript or added to an online repository such as Zotero.

**Response:**Three Tables and a Figure have now been uploaded with the manuscript under the title Supplementary Files (Supporting information).

2) Multiple testing should still be performed where appropriate, even though it will not change the conclusions, as its absence is distracting form the conclusion. **Response:** In the revision we have changed the statistical evaluations to now include a modified analysis that accounts for the influence of multiple testing on the reported associations, using the Bonferroni correction. The p value for a positive association has now been set at 0.005.

I also have the following minor corrections:
Line 66: Missing word? ”…, but the aetiology still remains poorly understood” for example ? **Response:** This has been corrected.

Line 78: Do you mean abundance above a certain level, or that abundance correlates with decreased risk of preterm birth?

**Response:** As mentioned above we have now removed from the text statements related to pregnancy outcome.

Line 82: “Whether” rather than “if”?

**Response:**This has been changed as requested.

Line 107: It is still unclear what is meant what is meant by “Suitability of the DNA for amplification”. I assume that it refers to lack of PCR inhibitors?

**Response:**In the revision we clarify the statement further. What we meant to convey and now explicitly state was that our ability to amplify the control gene indicates that the DNA was not degraded and there were no PCR inhibitors in the preparations.

Line 124: Please refer to original article using the method, since current reference also states “as previously described”. As far as I can see this is the correct reference. <https://doi.org/10.1038/s41598-017-09857-z> . Sequencing platform should still be stated in the abbreviated description.

**Response:** We thank the reviewer for these comments. We apologize for using an incorrect reference, This has now been corrected as suggested and we now also have increased our description of the protocol, including the sequencing platform utilized.

Reviewer #3: The data availability statement states that all data are included in the manuscript; however, in the letter to the editor the authors state ‘The raw data files are available upon request.“ Please clarify.

**Response:** We have now included all remaining experimental data as Supplementary files (supporting information).

Abstract
Line 32: L. iners was dominant: L. iners should be italicised, ‘were’ instead ‘was’

**Response:**These have been corrected.

“There was no association between TTV titre and pregnancy outcome.” However, you state in your letter to the editor that ‘most women at risk for preterm birth were treated with progesterone’ – If this is true, the authors cannot conduct this analysis and the conclusion is misleading.

**Response:** We completely agree and have now removed from the manuscript pregnancy outcome as an aim of our study. Please see our comment to the Editor above for a full explanation. Again, we wish to stress that the removal of pregnancy outcome data and their analysis does not reduce the value of our findings that the dominant bacteria in the vaginal microbiota and the concentration of compounds in vaginal fluid varies with the vaginal TTV titer. This novel finding furthers our understanding of differential bacteria domination in the vaginal microbiome in individual women and, hopefully, will eventually lead to development of protocols to deliberately alter the vaginal milieu to maximize women’s health.

“We conclude that quantitation of the TTV titer in vaginal secretions during the mid-trimester of pregnancy reflects the relative abundance of specific bacterial species and vaginal compounds at that site.” Please clarify that TTV titre was associated with relative abundance. Further, what is your conclusion? What does it meant that TTV titre is inversely or positively associated with specific bacteria?

**Response:** We thank the reviewer for these comments. Our conclusion is now been clarified to it more accurate and more easily understandable. “We conclude that variations in the TTV titer with the relative abundance of different bacterial species and concentration of vaginal compounds is consistent with the local immune status being an influence on vaginal fluid composition.”

Introduction
Line 63: or should not be italicized. **Response:** This has been corrected.

Line 83: I do not understand how the aims of the study was ‘to identify associations with pregnancy outcome“ if the authors included women who were treated with progesterone to avert PTB. Where these women still included in the analysis? Response: We completely agree. This statement has now been deleted. A complete explanation for doing this is provided above.

Methods
What were the enrolment criteria? If associations with pregnancy outcome were evaluated, these should be outlined in more detail. **Response:** Again, we have deleted from the text an analysis and discussion of pregnancy outcome. The enrolment criteria are now fully described in Methods.

Line 107-108: Please explain your control more. Beta-globin is a human gene while TTV is a virus. I am uncertain how this assessment would confirm stability of viral nucleic acids? **Response:**We have now expanded this section as follows. Suitability of the DNA for viral DNA amplification was evaluated by first testing the samples for amplification of the gene coding for beta-globin. This was successful in every case and indicated that the DNA was intact and that our preparations did not contain PCR inhibitors. In addition, specifically concerning the TTV DNA, we obtained a suitable amplification profile by real time PCR in every TTV positive samples and controls to validate that the viral DNA was quantitatively stable. This data is now included as a supplementary fig.1 (supporting information).

Line 124-127: Please expand this section. Which specific primers were used? Was data analysed using DADA2? Which database was used for taxonomic assignment? Which other downstream analysis was conducted? Were extraction controls included? How did the authors control for potential contamination? ?? **Response:** We thank the reviewer for these comments and for the opportunity to clarify this section. As mentioned in our response to the comment of Reviewer #1 we incorrectly referenced one of our subsequent studies and not our original reference. This has been corrected. Description of this analysis have now been greatly expanded. However, we would like to emphasize that details of our microbiome analyses have been already published and all relevant controls can be found in our included references.

Results:
Were p-values adjusted for multiple comparisons? The Spearman correlations will always be influenced by the large sample size – Just because a p-value is significant does not meant that a Spearman correlation of 0.1 biologically meaningful. **Response:** In the revision we now state in Methods that the p value for a positive association has been adjusted for multiple comparisons by employing the Bonferroni correction. The p value for a positive association is now set at <0.005.

Table 2: Bacterial species should be italicized. **Response:** This has been corrected.

Line 193-195: I do not think that this comparison is valid, given that some women in the cohort were treated when at high risk for PTB. **Response:** We again stress that when progesterone was given it was always **after** the collection of vaginal samples for analysis. Therefore, this treatment cannot influence our results.

Line 231: “In addition, the range of TTV titers that we identified in the vagina parallels the levels reported to be present in peripheral blood (2-8 log10 copies/ml), validating our TTV protocol.” How does this validate your protocol? Would you expect that systemic and vaginal viral titres are identical? **Response:** We agree that this statement was overly ambitious and not based on actual data. The sentence has now been deleted and replaced by the sentence, “It is still of interest to point out that the range of TTV titers we identified in the vagina parallel the levels reported to be present in peripheral blood (2-8 log_10_ copies/ml).

In summary, it is unclear to me what the manuscript in its current form adds to the literature. The study is correlative in nature, and the observed correlations are not particularly strong. Further, the assessment with birth outcomes seems to be inaccurate, given that some women received treatment to prevent PTB. **Response:** We completely agree, as discussed exhaustively above, that our assessment with birth outcomes may be inaccurate and, thus, this part of the manuscript has been deleted. However, we disagree that the manuscript does not substantially add anything new to the literature. Perhaps we did not stress strongly enough or clarify sufficiently the value of our observations. Specifically, the association between variations in the TTV titer and the relative abundance of specific bacterial species and vaginal compounds indicates that local changes in immune status likely influence vaginal fluid composition. This has now been made more explicit in the conclusion section of Discussion.
